# Development of Self-Sensing Textile Strengthening System Based on High-Strength Carbon Fiber

**DOI:** 10.3390/ma11102062

**Published:** 2018-10-22

**Authors:** Marcin Górski, Rafał Krzywoń, Magdalena Borodeńko

**Affiliations:** Department of Structural Engineering, Silesian University of Technology, ul. Akademicka 5, 44-100 Gliwice, Poland; rafal.krzywon@polsl.pl (R.K.); m.borodenko@onet.eu (M.B.)

**Keywords:** CFRP strengthening, textile sensor, strain gauge errors compensation

## Abstract

The monitoring of structures is one of the most difficult challenges of engineering in the 21st century. As a result of changes in conditions of use, as well as design errors, many building structures require strengthening. This article presents research on the development of an externally strengthening carbon-fiber textile with a self-sensing option, which is an idea is based on the pattern of resistive strain gauges, where thread is presented in the form of zig-zagging parallel lines. The first laboratory tests showed the system’s high efficiency in the measurement of strains, but also revealed its sensitivity to environmental conditions. This article also presents studies on the influence of temperature and humidity on the measurement, and to separate the two effects, resistance changes were tested on unloaded concrete and wooden samples. The models were then placed in a climatic chamber, and the daily cycle of temperature and humidity changes was simulated. The research results confirmed preliminary observations of resistivity growths along with temperature. This effect is more visible on concrete samples, presumably due to its greater amount of natural humidity. The strain measurement with carbon fibers is very sensitive to temperature changes, and applications of this method in practice require compensation.

## 1. Introduction

About 50 years ago, Concor and Owston [1] noted that most carbon fibers behaved in a simple manner of linear rises in resistance with strain. They also defined some of their important characteristics for measurements, such as the initial stress of fibers, time-dependency, and high strain rate or contact resistance [2]. Since these discoveries, the idea of self-sensing carbon fibers has been developed and is still being improved upon today. Currently, the development of two independent technologies using conductivity of carbon-based materials for strain measurement can be observed: printed gauges based on graphite carbon nanotubes [3,4,5,6,7], and those based on continuous carbon fiber [8,9,10,11,12,13,14,15,16,17]. It should be emphasized that only the latter allows for the use of an essential feature of carbon fibers, which is their high strength. In recent years, a form of technology enabling the 3D printing of carbon fiber sensors has also appeared [17,18], which should help in avoiding a quite laborious assembly process and should also improve sensor quality. Carbon fibers can also be used as light sensors [19].

Measurements performed using resistive strain gauges allow for investigation of changes in strain expressed by the change in resistance. Unfortunately, the measurement of the actual mechanical deformation overlaps with various effects which disturb the measurement signal. These effects mainly consist of environmental influences, such as thermal elongation of the measured object, temperature-dependent changes in strain gauge resistance, thermal shrinkage of the strain gauge measuring film, the thermal response of connecting wires, and surface conductivity with increasing humidity. Carbon-based materials are much more temperature-sensitive than conventional strain sensors made of constantan (copper-nickel alloy) [20]. The above factors may be influenced by the method of laying the wire, the material from which the strain gauge was made, and the ground material, as well as the type of adhesive that fixed the strain gauge. In the case of carbon fibers, their straightness may also be important [2,12], as well as the contact resistance [13] which may be limited by the matrix material, and the method of assembling the electrodes.

Typically, the effects of temperature and humidity is compensated for in the measurements by combining several strain gauges to form a half or full bridge. Through the skillful combination of strain gauges of the most commonly used Wheatstone bridge circuit [21], where strains from the measuring strain gauges and compensation strain gauges are reflected in the measurement signal with opposite signs (positive and negative), the bridge voltage only represents mechanical strains, and the temperature/humidity-dependent effects abolish each other.

Because identical, or even similar working conditions of measuring and compensating strain gauges may not always be possible, modern computational compensation systems which have been adapted to the thermal and moisture responses of strain gauges are increasingly being used today. In these types of measurements, a quarter-bridge circuit is sufficient, but knowledge of the temperate and humidity response functions is necessary.

This paper describes preliminary studies on the temperature-resistant dependence of externally strengthening carbon-fiber textile sensors which have been developed. These tests may be used in the future to build temperature-change compensation functions necessary for use in regard to future devices in engineering practice. They may also be used for development by the authors’ strengthening system, but it is believed that such self-monitoring solutions with reliable information about its deformation in various environmental conditions may be used for future smart structures made out of conductive fibers. 

## 2. The Idea of a Self-Sensing Strengthening System

Controlling the deformation of FRP (fiber reinforced polymer) composite fibers is much more important than the steels themselves traditionally used in constructions. This is due to their linear elasticity until destruction, which is usually sudden. For this reason, it is advisable to control the strains of the composite fibers to indicate excessive elongation, which may threaten their breaking or delamination.

As will be described in this paper, the system takes advantage of two features of carbon fibers, which are their superb strength and electrical conductivity. The idea of measuring strains by recording changes in the resistance is based on operation principles of strain gauges invented in 1938 by Edward E. Simmons and Arthur C. Ruge [22]. Such strain gauges contain a long, thin conductive wire arranged in a zig-zag pattern of parallel lines. The parallel orientation of wires multiplies the reading, which allows for an increase in the accuracy of the measurement of small strains. The same concept was used to create smart textiles. Carbon fiber tow, commonly used as a reinforcement for FRP strengthening systems, can also act as an electrical conductor. Deformation leads to changes in electrical resistance, allowing for self-monitoring of the fabric. The first tests have shown that changes in resistance for a single tow is too small compared to the technical abilities of the recording equipment used (e.g., the Wheatstone bridge). Increasing the length of the tow by its wiggly lead (a copy of the zig-zag pattern of the strain gauge) significantly improved the sensitivity of the system. 

**Figure 1:**
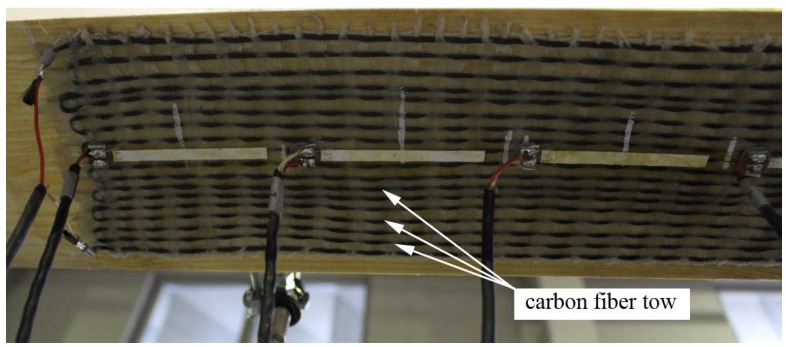
The second generation of the textile sensor, adhered to a specimen of timber.

In this way, the first prototype was tested on small models of concrete slabs. To verify the accuracy of the measurement, the device was accompanied by paper strain gauges adhered along its length (Figure 1). A more detailed description of these studies is described in [23]. These initial trials confirmed the capabilities of developed smart textiles. However, the sensitivity of the device still proved to be insufficient, especially for measuring small changes in the strain. A modification allowing for an increase in the accuracy of the measurement was the introduction of two parallel threads of carbon fibers placed end to end. When the measuring bridge was connected to the two ends of independent threads, the measured resistance increased, because besides the previous volume resistance (growing with the elongation of carbon fiber), the phenomenon of contact resistance appeared at the interface of parallel fiber threads. Despite the controversy, which was mainly related to the accuracy of the measurement of large deformations at which micro-cracks may appear, and with them, rapid increases in contact resistance, the results of the first tests turned out to be quite promising. The developed textile sensor was checked in regard to whether it could act as an independent external reinforcement and as a local strengthener of existing reinforcements. The strengthening effectiveness which was achieved can also be assessed by using a single carbon sheet layer while maintaining the ability to measure strains until their failure. The results of these studies are presented in Reference [24].

The production method described was based on traditional plain weaving, where alternating carbon and acrylic threads were arranged as a warp and stabilized by the weft made of cotton. However, this technology has several significant disadvantages. The carbon fiber must be continuous and well-stressed during weaving, which may hinder the automation of production, and only the classic weaving loom can be used. The fiber types used also makes the textile sensor soft, and although this facilitates its application, after adhering, the carbon fibers may not be sufficiently stressed, which can worsen the strengthening efficiency and change the gauge factor. Specifying the gauge factor value is also one of the biggest drawbacks of the developed solution, which is why paper strain gauges were used for the trial tests. We also observed that resistance not only depended largely on the total length of the carbon fiber thread, but also on the lamination conditions. This is most likely related to the change in contact resistance conditions.

**Figure 2:**
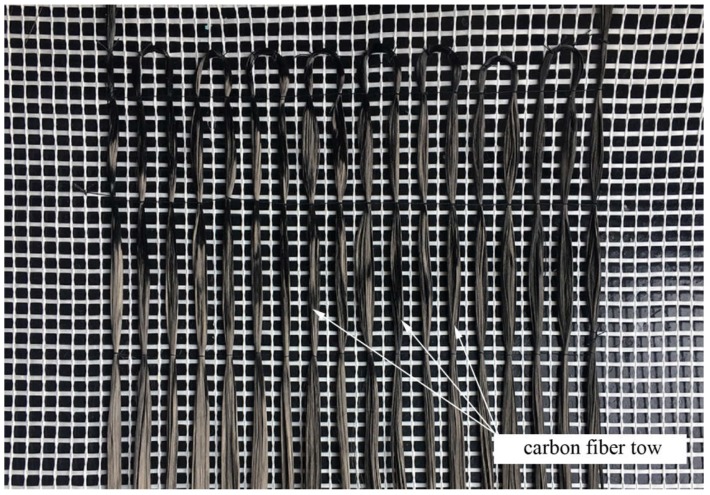
The third generation of the textile sensor during fabrication.

The third generation of the intelligent textile is currently being tested. The concept of the parallel arrangement of a double-fiber thread was abandoned, which means that the volumetric resistance is now directly responsible for the measurement. However, the main modification is in regard to the production method. Weaving was replaced by fiber stabilization by fastening to the composite mesh matrix, and for this purpose, a glass fiber plaster-reinforcing mesh was used. The dry carbon thread was stitched to the mesh strands, as shown in Figure 2. This stabilization method reduces the risk of accidental short-circuiting during the assembly of the sensor, and there is no need for additional separating acrylic threads. The sensor can be manufactured even in construction-site conditions, and no machines are needed, such as a weaving loom. The change in the resistance of this type of sensor should depend mainly on the total length and cross-section of the carbon fibers; therefore, its gauge factor should be easier to determine.

## 3. Tests of the Influence of Temperature on the Resistance

The research described in this chapter is concerned with two types of sensors: the woven sensor, which was the second generation, adhered to the wooden substrate; and stabilized mesh, the latest generation, adhered to the concrete substrate. In our tests, two-component epoxy S&P Resin 55HP was used, which is an adhesive intended for fixing carbon sheets onto various types of substrate. Wet lay-up technology was also applied according to the following method: firstly, the substrate was impregnated, then the sensor was placed, and the carbon fibers were aligned and saturated with resin. The adhesive layer was leveled out with a roller.

Table 1 presents the basic parameters of both types of sensors. The wooden sample was made of pinewood, and dimensions of the prism were 1200 × 158 × 78 mm. The wood fibers and sensor thread were parallel. The thermal expansion coefficient along the fibers of pinewood was equal at around 3 × 10^−6^/°C. The precast concrete sample was made of plain concrete, and was 1000 mm long and 200 × 60 mm for the cross-section. The coefficient of the thermal expansion of concrete was equal to 12 ×10^−6^/°C.

**Figure 3:**
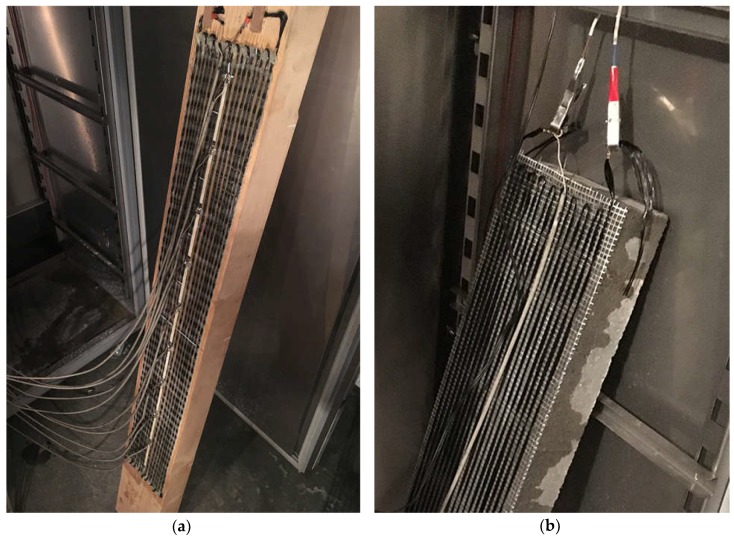
Samples inside the climate chamber during the test: (**a**) second generation of the sensor and timber sample; (**b**) third generation of the sensor and concrete sample.

The models were tested after more than a month of curing the adhesive. They were stored in a stable temperature of about 20 °C and 60% humidity two hours before the test samples were placed in a climatic chamber (Figure 3). 

Due to the preliminary nature of the research, tests for both samples were provided in four different cycles of temperature change with a small variation in humidity, which are the following:

- heating from +20 °C to +40 °C with a simultaneous humidity change from 60% to 65%,
- heating from +20 °C to +60 °C with a simultaneous change in humidity from 60% to 65%,
- cooling from +20 °C to 0 °C with a simultaneous reduction of humidity from 60% to 55%,
- cooling from +20 °C to −20 °C with a simultaneous reduction of humidity from 60% to 55%.

Concrete and wooden samples were tested separately. Before the next test cycle, they were stored for at least two days in the above-mentioned stable conditions to regain their natural humidity and temperature.

After being placed in a climatic chamber, a control measurement was carried out using a simple ohmmeter to check initial resistance and to exclude accidental short circuits. Then, the textile sensor was connected to the Wheatstone bridge, the chamber was closed, and the test commenced. 

Although the climatic chamber allows for simultaneous changes in temperature and humidity, the temperature remains the priority. It should be also emphasised that increases and decreases in temperature are not linear; in the first phase there is an intense change, followed by a phase of slow adjustment to the expected temperature. This property causes the measurement paths in similar temperature ranges to vary in cycles with different limit values.

During the test, the actual temperature and humidity were saved by the chamber controller, and strains simultaneously expressed by resistance changes were recorded every 60 s through a computer connected to the Wheatstone bridge. This measurement corresponded to the sum of the actual thermal deformation of the sample and apparent deformation, which expresses the wanted change in the sensor’s resistance under the influence of temperature and humidity.

## 4. Test Results

**Figure 4:**
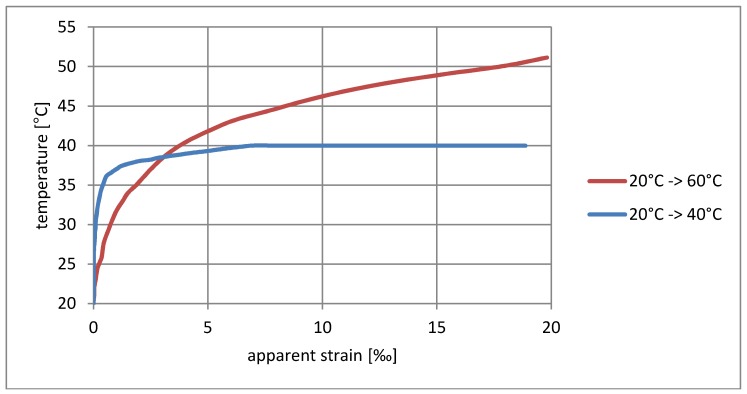
Dependence of apparent strain on rising temperature for the wooden sample.

**Figure 5:**
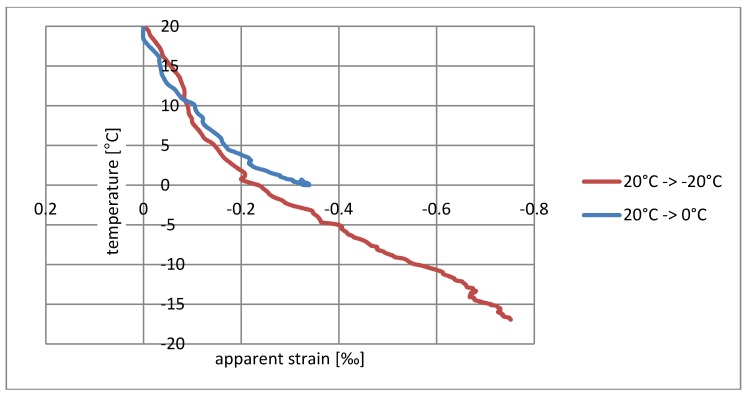
Dependence of apparent strain on decreasing temperature for the wooden sample.

**Figure 6:**
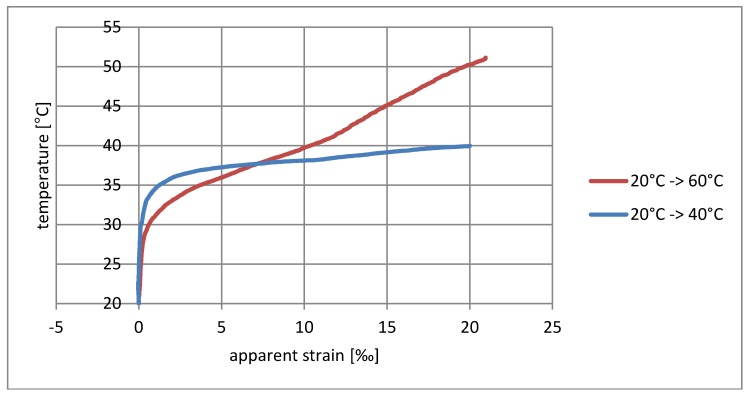
Dependence of apparent strain on rising temperature for the concrete sample.

**Figure 7:**
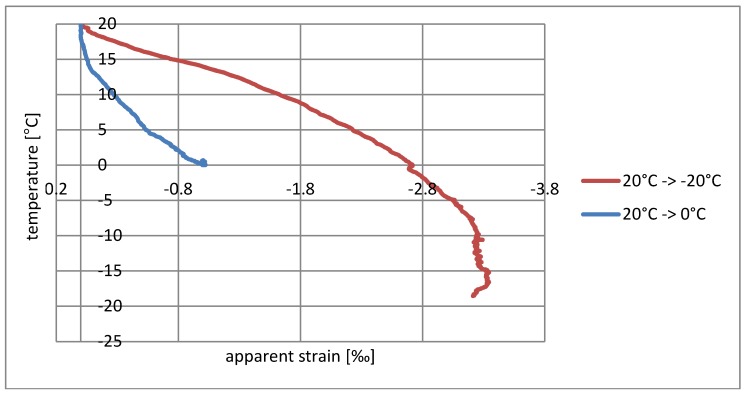
Dependence of apparent strain on decreasing temperature for the concrete sample.

The influence of temperature and relative humidity on the resistance measurement must be considered simultaneously, and it should also be noted that the relative humidity itself is temperature-dependent. To simplify the interpretation of the results, measures were provided for relatively small changes in relative humidity and the results are presented only as a function of temperature. They are shown in diagrams as having a form of dependence on temperature changes on the apparent deformation measured. Figure 4 and Figure 5 show the test results for the sensor on a wooden sample, and Figure 6 and Figure 7 on a concrete sample. The situation of the rising temperature and the decreasing temperature is shown independently in separate diagrams.

## 5. Discussion

The measured strain consists of a real elongation of the sample as a result of thermal expansion and apparent strain resulting from the temperature sensitivity of the textile sensor. The direction of observed resistance changes is correct. The increase in temperature is indicated as elongation, while the temperature decrease is indicated as shortening. If the changes shown were purely the result of thermal deformability, then for wood the measured deformation should not exceed 0.003‰/°C, whereas for concrete it should be 0.012‰/°C; however, the obtained strains are much greater, and the temperature–strain relationship is also not linear.

The sensor’s tendency to indicate apparent strain is particularly visible for rising temperatures. Considering the elasticity coefficient of carbon fibers, the measured strain > 20‰ (Figure 4 and Figure 6) theoretically corresponds to a stress of 5.4 GPa, thus breaking the carbon fibers in practice. The situation is slightly better in the case of decreasing temperatures’ ‰, where the error for wood does not exceed 0.75‰ (Figure 5), while for concrete it is 3.3‰ (Figure 7). This difference can also result from a slightly different type of textile sensor used in both cases. However, a faster increase in the conductivity of concrete samples may also result from humidity changes on their surface—due to the better thermal conductivity, the concrete base can promote condensation of water vapor. The indicated value of apparent strain shows the impact of compensation due to temperature changes on the result. The tendency for there to be such a high inflation of the result when the temperature rises means that the system is completely unusable, even for approximate estimations of stress changes. 

**Figure 8:**
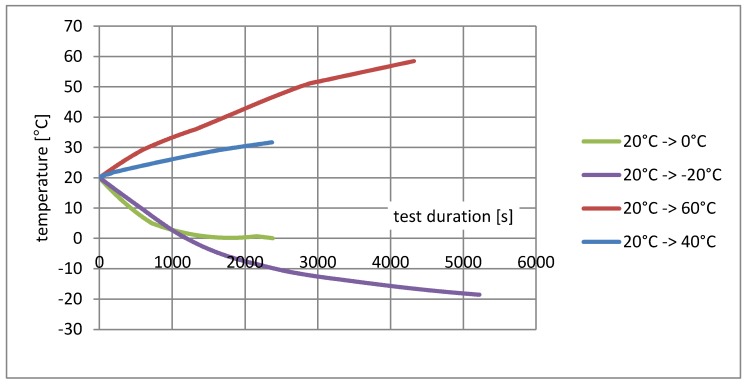
Paths of the temperature change during heating or cooling inside the climate chamber.

Another interesting observation is related to the speed of temperature changes. Heating tests from 20 °C to 40 °C and 20 °C to 60 °C, as well as cooling tests from 20 °C to 0 °C and 20 °C to −20 °C were running at different rates (shown in Figure 8). The paths of strain increments in the same temperature ranges shown in the diagrams (Figure 4, Figure 5, Figure 6 and Figure 7) are totally different. At the current level of research, it is difficult to explain the reason for this phenomenon. It may be associated with the evaporation of water from the surface of the material, which would explain the fastest increase in resistance in the first phase of heating. To confirm this theory, further research is needed for different rates of temperature change in various ranges. 

The problem of the influence of humidity, including its condensation, also needs to be solved. The physical mechanisms of the sensor’s high sensitivity to temperature changes also require clarification. Perhaps it will be necessary to modify the concept of a developed textile sensor, such as by introducing an additional isolating layer that cuts off carbon fibers from moisture.

## 6. Conclusions

Previous research on self-monitoring smart textiles made out of carbon fiber [23,24] demonstrated the problem of the effects of the environment on the observed results. Former tests were conducted in stable environmental conditions, while the self-monitoring strengthening system is expected to be built into existing building structures as bridges, which will be affected by various levels of temperature and moisture during their performance. 

It is thus crucial to define the relation between the readings of the structure’s deformation under the load with changing environmental conditions. 

Due to very few studies on this issue, the research team undertook steps to describe this phenomenon and to provide reliable tools for the determination of real strain situations in different temperatures and humidity levels.

The presented tests are only preliminary and do not allow a full determination of the relationship between temperature and resistance change. However, even such limited research shows the importance and complexity of the problem. Resistance changes are not only a function of temperature changes, but also the speed of these changes or the type of substrate. Caused by increasing temperature, an indication error exceeding 5.4 GPa clearly illustrates the importance of the development of measurement methods using carbon fibers, and the development of effective methods to compensate for the changes in temperature. 

Authors believe that this research path may be important not only for developed smart strengthening systems, but also for other conductive fiber-based smart structures for various branches of the industry. 

## Figures and Tables

**Table 1:** Properties of the textile sensor.

Sensor Generation	Woven (2nd)	Mesh Stabilized (3rd)
construction	Two parallel threads of conductive carbon fiber, separated by a thread of acrylic fiber (1.17 g/cm^3^) and stabilized by cotton weft (1.54 g/cm^3^)	A single thread of conductive carbon fiber fastened to the glass fiber plaster-reinforcing mesh (8 × 8 mm; 145 g/cm^3^)
number of loops	18	18
length of the sensor	1000 mm	1000 mm
rowing	2 × 24,000 filaments/thread 2 × 1600 tex	24,000 filaments/thread 1600 tex
strength of carbon fiber	5000 MPa	
modulus of elasticity	270 GPa	
elongation at break	1.9%	
filament resistivity	14 μΩm	
initial resistance	174.8 Ω ^1^	212 Ω
gauge factor	1.24	1.19

^1^ Before lamination of fibers.
